# Phylogenetic Structure of *Synechococcus* Assemblages and Its Environmental Determinants in the Bay and Strait Areas of a Continental Sea

**DOI:** 10.3389/fmicb.2022.757896

**Published:** 2022-04-06

**Authors:** Ting Wang, Xi Chen, Jialin Li, Song Qin

**Affiliations:** ^1^Key Laboratory of Coastal Biology and Biological Resource Conservation, Yantai Institute of Coastal Zone Research, Chinese Academy of Sciences, Yantai, China; ^2^College of Environmental Science and Engineering, Ocean University of China, Qingdao, China; ^3^CAS Key Laboratory for Experimental Study Under Deep-Sea Extreme Conditions, Institute of Deep-Sea Science and Engineering, Chinese Academy of Sciences, Sanya, China; ^4^University of Chinese Academy of Sciences, Beijing, China; ^5^College of Marine Life Science, Ocean University of China, Qingdao, China; ^6^Center for Ocean Mega-Science, Chinese Academy of Sciences, Qingdao, China

**Keywords:** picoplankton, cyanobacteria, genetic diversity, phylogeny, marginal sea, silicon accumulation, high-throughput sequencing

## Abstract

Marine *Synechococcus*, a significant contributor to primary production, shows high phylogenetic diversity. However, studies on its phylogenetic composition in the Bohai Sea, the largest continental sea in China, are lacking. We sequenced *rpo*C1 (encodes the RNA polymerase β’ subunit protein) in samples from the Laizhou Bay (LZB) and Bohai Strait (BS) in June and November using high-throughput sequencing to reveal the phylogenetic composition of *Synechococcus* assemblages in the bay and strait areas of the Bohai Sea. In total, 12 lineages representing *Synechococcus* subclusters S5.1, S5.2, and S5.3 were identified. Spatially, clade I was obligately dominant in BS. In contrast, the *Synechococcus* assemblage in LZB was more diverse, with clades VI and III being highly abundant. In addition, we detected strong variation in *Synechococcus* structure between June and November in the Bohai Sea. Clades II, III, XX, and miyav were only detected in November. Vertically, variation in *Synechococcus* assemblage was not apparent among the water layers probably due to the shallow water depth with intense water mixing. Results of redundancy analysis (RDA) and random forest (RF) analysis together highlighted the key role of silicate in the *Synechococcus* assemblage. Our results suggested that the Bohai Sea provides various niches for different *Synechococcus* clades, resulting in a special phylogenetic composition of the *Synechococcus* assemblage, compared with that in the adjacent shelf sea and other continental seas in the world.

## Highlights

- *Synechococcus* composition varied spatially between Laizhou Bay (LZB) and Bohai Strait (BS) in Bohai Sea.
- *Synechococcus* clades II, III, XX, and miyav were detected only in November.
- Vertical variation in *Synechococcus* assemblage was not apparent.
- Silicate played a key role in the *Synechococcus* assemblage in this continental sea.

## Introduction

Marine *Synechococcus*, a widely distributed autotrophic picoplanktonic organism, plays a key role in the euphotic zone of global oceans in terms of primary production and carbon fluxes (Flombaum et al., 2013; Paulsen et al., 2016). The phylogenetic diversity of *Synechococcus* has been described using a range of conserved markers, such as 16S rRNA (Dufresne et al., 2008), ITS (16S-23S internally transcribed spacer; Ahlgren and Rocap, 2006; Huang et al., 2012), *rpo*C1 (subunit of RNA polymerase; Xia et al., 2017a), *ntc*A (a nitrogen regulatory gene; Penno et al., 2006), and *pet*B (cytochrome b6) genes (Mazard et al., 2012). Among these gene markers, housekeeping gene of *rpo*C1 is single copy in all known *Synechococcus* genomes and has higher genetic resolution than the 16S rRNA gene (Mühling et al., 2006), so it has been widely used in studies involving the genetic diversity of *Synechococcus* (Xia et al., 2017a). So far, marine *Synechococcus* is classified into three subclusters: S5.1, S5.2, and S5.3 (Ahlgren and Rocap, 2012). In each subcluster, various clades can be further distinguished. S5.1 shows the highest phylogenetic diversity, consisting of at least 20 clades including clades I to XVI, CRD1/2, ENV-B/C, and so on (Choi et al., 2013a).

Studies targeting the global distribution of *Synechococcus* assemblages have revealed clear spatial partitioning for the main *Synechococcus* lineages (Farrant et al., 2016; Sohm et al., 2016; Xia et al., 2019; Li et al., 2021). The current notion regarding the biogeography of *Synechococcus* lineages indicates that S5.1 clades I and IV are most abundant in colder and eutrophic waters (Kent et al., 2019). S5.1 clades II, III, V, VI, and VII appear frequently in tropical and subtropical waters, while clades CRD1 and ENV-B/C dominate upwelling zones with low iron content (Zwirglmaier et al., 2008; Post et al., 2011; Sohm et al., 2016). In addition, the abundance and structure of *Synechococcus* assemblages also change with seasons (Tai and Palenik, 2009; Tai et al., 2011). In the coastal Pacific Ocean, for example, *Synechococcus* clade IV was prevalent throughout the year, while clades II and III appeared only in late summer and winter (Tai and Palenik, 2009).

The phylogenetic diversity of the *Synechococcus* community has been well studied in the South China Sea and East China Sea (Ma et al., 2004; Choi and Noh, 2009; Jing and Liu, 2012; Choi et al., 2013b; Chung et al., 2015). Furthermore, it has been studied in subtropical estuarine waters and coastal waters, such as Hong Kong estuary and coast, Pearl River estuary, and Changjiang River estuary (Jing et al., 2009b; Chung et al., 2015; Xia et al., 2015, 2017a). However, relevant studies in sea areas at higher latitudes in China are scarce (Li et al., 2021). In particular, little is known about the community composition of, and environment driven by, *Synechococcus* assemblages in the Bohai Sea, the largest continental sea of China. In fact, a few studies have focused on the population structure of *Synechococcus* in the continental seas in the world. So far, such studies have been conducted in limited continental seas, such as the Sea of Okhotsk (Jing et al., 2009a), Mediterranean Sea (Mella-Flores et al., 2011), and Red Sea (Fuller et al., 2005). The hydrologic characteristics of these regions are significantly influenced by continents, including the input of continental rivers, the human activity along the coasts, etc (Wang et al., 2018a; Temino-Boes et al., 2019). It is of great significance to investigate the phylogenetic composition and niche division of *Synechococcus* assemblages in these sea areas with anthropic environmental conditions.

Bohai Sea enjoys monsoon climate of medium latitudes with clear seasonal climate change. It is connected to the Yellow Sea only through the Bohai Strait. Serious anthropogenic interference and bad water quality exchange have resulted in poor water conditions in the Bohai Sea (Zhang et al., 2015). The bay areas, especially the Laizhou Bay, are polluted because of the discharge of large amounts of industrial wastewaters, domestic sewage, and wastewater from animal raising industries (Lü et al., 2015). The inflow of continental rivers, especially the Yellow River (the second largest river in China), also influences the biology in these areas. In contrast, BS has better water quality conditions owing to water exchange with the northern Yellow Sea. *Synechococcus* generally dominated the total cell abundance in most seasons in the above regions (Yan et al., 2020; Wang et al., 2021). However, the phylogenetic diversity of *Synechococcus* in this region has not been investigated. We collected samples from the Laizhou Bay and Bohai Strait of Bohai Sea in early summer and winter to investigate the population composition and effect of the environment on *Synechococcus* clades based on *rpo*C1 high-throughput sequencing, which is of great importance for understanding the niche division and population shift of *Synechococcus* assemblages seriously affected by human activities.

## Materials and Methods

### Sampling

Field sampling was conducted at stations LZB and BS in the Bohai Sea onboard the Research Vessel “Chuangxin I” in June and November 2019 (Table 1; Supplementary Figure S1). Station LZB is located in Laizhou Bay, which is seriously affected and polluted by human activities and the inflow of continental rivers. In comparison, station BS located in Bohai Strait has water exchanges with the North Yellow Sea, and the water quality is better. At each station, samples were collected from three or four standard depths according to the specifications for the oceanographic survey (National Standard of China, GB/T 12763-2007). Seawater was collected in Niskin bottles carried by a CTD rosette sampler (Sea-Bird 911Plus, Sea-Bird Electronics Inc., Bellevue, WA, United States). For DNA extraction, 1,000 ml of seawater was sequentially filtered through 48-μm nylon mesh (to remove microphytoplankton) and 0.22-μm polycarbonate filters (Millipore Co., Bedford, MA, United States). Then, the collected filters were flash frozen in liquid nitrogen and stored at −80°C until further analysis.

**Table 1:** Information of sampling station, latitude, longitude, date, and depth of all samples.

Sample name	Sampling station	Latitude (°N)	Longitude (°E)	Sampling date	Sampling depth (m)
BS-0-S	BS	38.5	121.24	June 10, 2019	0
BS-10-S					10
BS-30-S					30
BS-B-S					53
LZB-0-S	LZB	37.75	119.92	June 16, 2019	0
LZB-10-S					10
LZB-B-S					15
BS-0-W	BS	38.51	121.24	November 6, 2019	0
BS-10-W					10
BS-30-W					30
BS-B-W					53
LZB-0-W	LZB	37.8	119.93	November 8, 2019	0
LZB-10-W					10
LZB-B-W					15

BS, Bohai Strait; LZB, Laizhou Bay; B, bottom water layer; S, summer (sampled in June); and W, winter (sampled in November).

### Measurement of Environmental Variables

Temperature, salinity, depth, pH, turbidity, dissolved oxygen, and concentration of chlorophyll *a* (chl. *a*) were measured *in situ* using the CTD (Sea-Bird 911Plus, Sea-Bird Electronics Inc., Bellevue, WA, United States). The nutrient concentrations, including nitrite (NO_2_^−^), nitrate (NO_3_^−^), ammonium (NH_4_^+^), phosphate (PO_4_^3−^), and silicate (SiO_4_^4−^) were measured using standard colorimetric methods with an AA3 segmented flow analyzer (Seal Analytical GmbH, Germany; Dafner, 2015).

### DNA Extraction, PCR, and Sequencing

Each polycarbonate membrane filter was disrupted by bead-beating method and then taken for total genomic DNA extraction using the Fast DNA SPIN Kit (MP BIO, United States; Li et al., 2021). The DNA concentrations were determined using a Nano Drop 2000c spectrophotometer (Thermo Fisher, United States). PCR was performed in the thermocycler *GeneAmp®* PCR system *9700* (Perkin Elmer, United States) in triplicate (Li et al., 2019) with primers *rpo*C1-39F (5′-adaptor+barcode+GGNATYGTYTGYGAGCGYTG-3′) and *rpo*C1-462R (5′-adaptor+CGYAGRCGCTTGRTCAGCTT-3′; Mühling et al., 2006). All reactions were performed in 20 μl containing 4 μl of 5× FastPfu Buffer, 2 μl of 2.5 mM dNTPs, 0.8 μl of 5 μM forward and reverse primers, 0.4 μl of FastPfu Polymerase, 0.2 μl of BSA, 10 ng template DNA, and ddH_2_O. Thermal cycling protocol was as follows: following an initial denaturation step of 95°C for 3 min, 37 cycles at 95°C for 30 s, 53°C for 30 s, and 72°C for 45 s, followed by a final extension step at 72°C for 10 min. The triplicate amplified products were combined in one, and then it was gel-purified using the Qiaquick gel purification kit (Qiagen, Hilgen, Germany) as described by the manufacturer. The purified amplicon was roughly quantified with agarose gel imaging by QuantiFluor-ST Fluorometers (Promega, Madison, WI, United States). The amplicons obtained were sequenced by the Majorbio Co., Ltd. (Shanghai, China) using Illumina MiSeq sequencing platform (PE300).

### Processing of High-Throughput Sequencing Data

QIIME2 was used to process raw sequence profiles as previously described with several modifications (Caporaso et al., 2010). First, the raw data were quality controlled to retain high-quality reads with criterions of Phred quality score > 20, no ambiguous bases, and consecutive high-quality bases >80% of total read length (Brown et al., 2017). USEARCH was used to filter chimeras (Edgar et al., 2011). Then operational taxonomic units (OTUs) were calculated at 95% similarity. OTU representative sequences were first identified using Basic Local Alignment Search Tool (BLAST) against the nt database, i.e., partially non-redundant nucleotide sequences National Center for Biotechnology Information (NCBI) database (Zhang et al., 2000), and those that do not belong to cyanobacteria are discarded. Representative sequences with relative abundance > 0.1% were extracted and aligned with the reference sequences. Modeltest and maximum likelihood phylogenetic tree construction were performed using Mega 7.014 with the model GTR + G + I and 200 bootstraps (Xia et al., 2017a). All sequences obtained from this study have been deposited in the NCBI Sequence Read Archive (SRA) under the accession number PRJNA753275.

### Statistical Analysis and Visualization

Ocean Data View (ODV v. 5.1.7) was used to sketch the map of sampling stations (Schlitzer, 2002). The package *corrplot* in R Language v. 3.6.1 was used to calculate Spearman’s correlation (Tai and Palenik, 2009). A *p*-value less than 0.05 was considered statistically significant. The package *pheatmap* in R Language v. 3.6.1 was used to draw the heatmaps with sample clustering. The package *vegan* in R Language v. 3.6.1 was used to calculate alpha diversity indices, beta diversity distance, as well as redundancy analysis (RDA; Dixon, 2003). Statistical Product and Service Solutions (SPSS v. 17.0) was used to test the significance of differences in alpha diversity indices among samples at different stations and in different months through ANOVA with a *post hoc* test (Tukey test; George, 2011). A *p*-value less than 0.05 was considered statistically significant. The principal coordinate analysis (PCoA) ordination was conducted at the OTU level using the package *vegan* in R Language v. 3.6.1 (Dixon, 2003). Dissimilarity in community structure among samples at different stations and in different months was assessed with permutational multivariate ANOVA (PERMANOVA) using 999 permutations for significance testing (Anderson, 2017). Before RDA, environmental variables with variance inflation factor (VIF) more than 10 were filtered out. VIF is an indicator that tests the multicollinearity of regression analyses (Mansfield and Helms, 1982). VIF more than 10 represents that the corresponding variables provide little independent explanatory ability and need to be deleted in the calculation of regression analyses (Franke, 2010). The package *randomForest* in R Language v. 3.6.1 was used to conduct random forest (RF) analysis (Liaw and Wiener, 2002). The package *psych* in R Language v. 3.6.1 was used to characterize properties of co-occurrence networks (Revelle and Revelle, 2015). Then Gephi v0.9.2 was used to draw the network (Bastian et al., 2009). The hubba (key) score in each network was calculated using a plugin, CytoHubba, in the Betweenness method in software Cytoscape v3.8.0 (Chin et al., 2014). Finally, the package *basicTrendline* in R Language v. 3.6.1 was used to conduct linear ordinary least square (OLS) regression (Mei et al., 2018). OLS regression is a statistical method that estimates the relationship between the variables by minimizing the sum of squared differences between the observed and predicted values of the dependent variable (Hutcheson, 1999).

## Results

### Seawater Environmental Conditions

**Figure 1:**
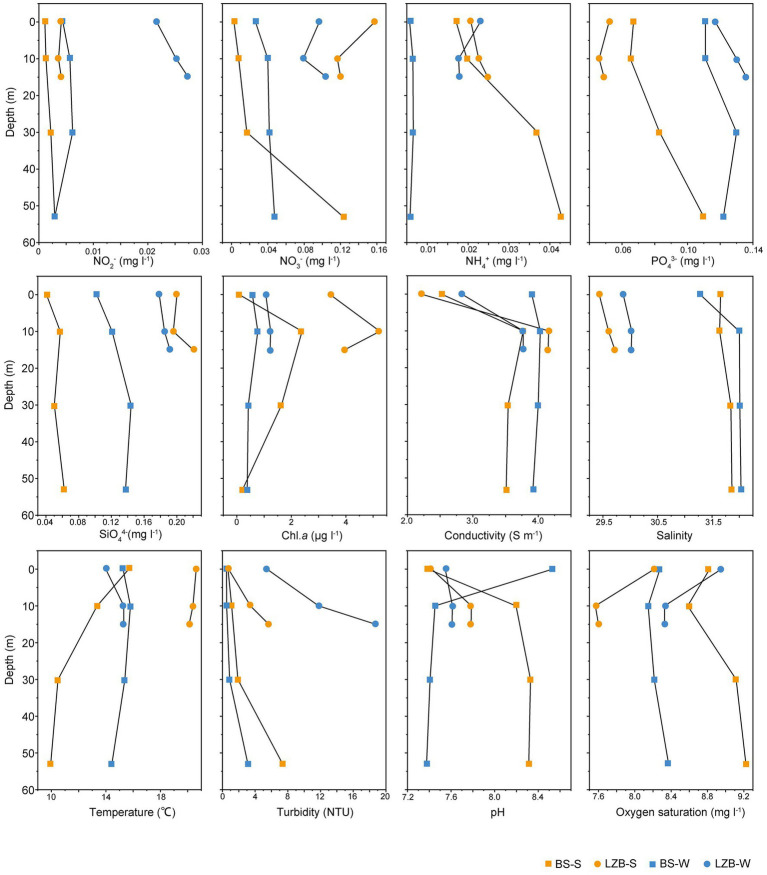
Environmental variables in the studied stations of Bohai Sea.

Environmental parameters of stations LZB and BS in June and November are shown in Figure 1. The highest mean values of nitrate (NO_3_^−^), silicate (SiO_4_^4−^), chl. *a*, and temperature were detected in LZB-S, i.e., samples at station LZB in summer (June). In contrast, the highest mean values of nitrite (NO_2_^−^), phosphate, and (PO_4_^3−^) concentrations, and turbidity appeared in LZB-W, i.e., samples at LZB in winter (November). The salinity was high in both June and November at the BS station. Hierarchical clustering of samples in the heatmap based on scaled environmental data showed that samples at station BS were separated from those at LZB (Supplementary Figure S2). Besides, the environmental condition between samples in June and November was usually different, no matter what station, BS or LZB. However, the environmental condition of samples in different water layers at the same stations and in the same month was very similar.

There was no significant correlation between environmental variables and water depth, indicating good water mixing among water layers (Supplementary Figure S3). However, multiple significant correlations were observed among the variables. In particular, the concentration of SiO_4_^4−^ significantly correlated with six variables, including the concentration of NO_2_^−^ (*ρ* = 0.68, *p* < 0.01) and NO_3_^−^ (*ρ* = 0.67, *p* < 0.01), temperature (*ρ* = 0.6, *p* < 0.05), turbidity (*ρ* = 0.55, *p* < 0.05), salinity (*ρ* = −0.79, *p* < 0.01), and oxygen saturation (*ρ* = −0.59, *p* < 0.01).

### Community Characteristics of *Synechococcus* Assemblages

High-throughput sequencing of *rpo*C1 generated 431,129 quality sequences from 14 samples (Table 2). The Good’s coverage for the observed OTUs was >99%, indicating a near-complete sampling of the community. The Shannon diversity index ranged from 0.60 to 2.28, with an average of 1.81, and the Chao1 estimator ranged from 17 to 68, with an average of 41. On average, community diversity, represented by the Shannon diversity index and community richness represented by the Chao1 estimator of *Synechococcus* assemblage, were higher in winter than that in summer. Geographically, average community diversity and richness of *Synechococcus* assemblage were higher in LZB than in BS. Among all variables, only pH showed significant correlation with Shannon diversity index (*ρ* = −0.78, *p* < 0.01) and Chao1 estimator (*ρ* = −0.86, *p* < 0.01).

**Table 2:** High-throughput sequencing information and alpha diversity parameters.

Sample	Sequence numbers	Sequence mean length	Shannon diversity index	Chao1 estimator
BS-0-S	23,496	395	2.15	52
BS-10-S	43,658	400	1.39	18
BS-30-S	56,798	403	1.22	17
BS-B-S	14,515	392	1.55	39
LZB-0-S	10,502	365	1.75	43
LZB-10-S	14,202	368	1.73	42
LZB-B-S	5,828	361	2.07	33
BS-0-W	57,023	402	0.60	30
BS-10-W	36,962	387	2.28	68
BS-30-W	35,270	401	2.06	51
BS-B-W	36,232	399	2.25	53
LZB-0-W	24,061	395	2.00	47
LZB-10-W	21,324	379	1.96	33
LZB-B-W	51,258	397	2.27	51

**Figure 2:**
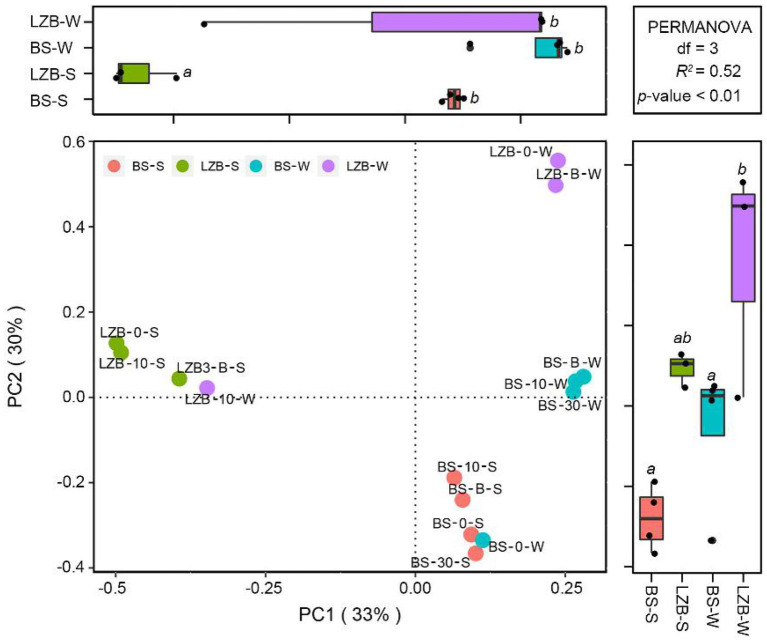
The principal coordinate analysis (PCoA) with permutational multivariate ANOVA (PERMANOVA) test showing the variation in *Synechococcus* assemblages at operational taxonomic units (OTU) level among samples at different stations and in different months. BS-S, samples at station Bohai Strait (BS) in summer (June); BS-W, samples at station BS in winter (November); LZB-S, samples at station Laizhou Bay (LZB) in summer (June); LZB-W, samples at station LZB in winter (November).

The PCoA with the PERMANOVA test demonstrated that along the axis PC1, *Synechococcus* assemblages in LZB-S were significantly different from those in other samples (*p* < 0.01; Figure 2). Along the axis PC2, *Synechococcus* assemblage in BS-S, i.e., samples at station BS in summer (June), and BS-W, i.e., samples at station BS in winter (November), were significantly different from those in LZB-W (*p* < 0.01). In total, axes PC1 and PC2 explained 63% of the community variation of *Synechococcus* assemblage.

### Community Composition of *Synechococcus* Assemblages

**Figure 3:**
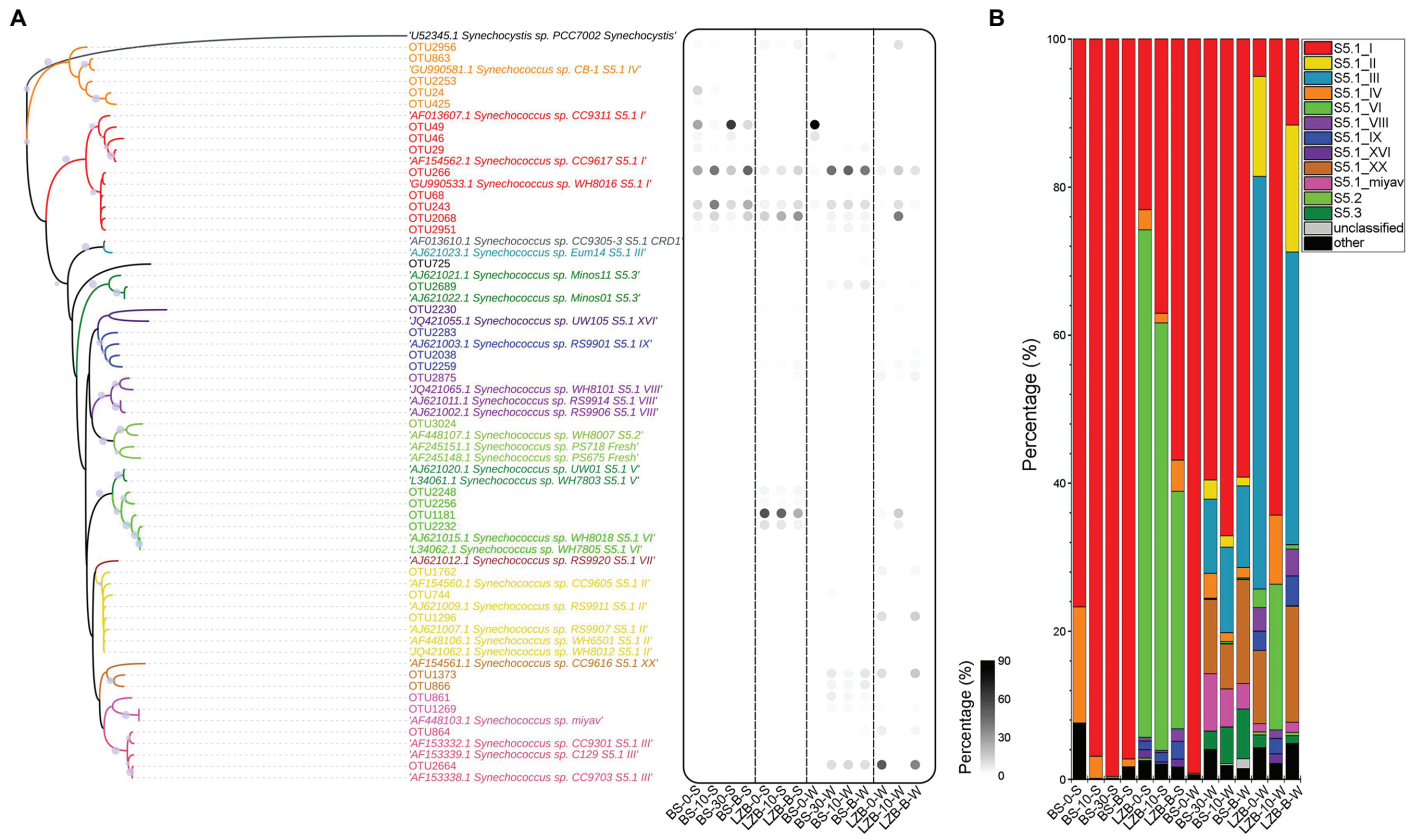
Genomic composition of *Synechococcus* assemblages. **(A)** The maximum-likelihood (ML) tree constructed using *rpo*C1 sequences with relative abundances of more than 0.1%. **(B)** The relative abundance of *Synechococcus* clades.

The maximum-likelihood (ML) tree constructed using *rpo*C1 sequences showed that all *Synechococcus* subclusters, S5.1, S5.2, and S5.3, were well identified, in which *Synechococcus* S5.1 was the dominant subcluster and could be further divided into 10 clades (Figure 3A). The percentage of *Synechococcus* S5.2 and S5.3 in the *Synechococcus* genotype of the studied area was negligible, accounting for only 0.10 and 1.21% on average, respectively. Among *Synechococcus* S5.1, clade I was the most preponderant clade, accounting for 60.98% of the clades in all samples. *Synechococcus* clade VI increased sharply in LZB-S and *Synechococcus* clade III was abundant in November, especially at depth of 0 m (LZB-0-W) and at the bottom of (LZB-B-W) layers of LZB-W. *Synechococcus* S5.1 clades I, IV, VI, VIII, IX, and XVI, S5.2, and S5.3 were detected in both months, while *Synechococcus* S5.1 clades II, III, XX, and miyav were only found in November (Figure 3B). In addition, in BS-S, a mass of *Synechococcus* clades, including clades II, III, VI, VIII, IX, XVI, XX, and miyav, were absent.

### The Co-occurrence Patterns of *Synechococcus* Genotypes

**Figure 4:**
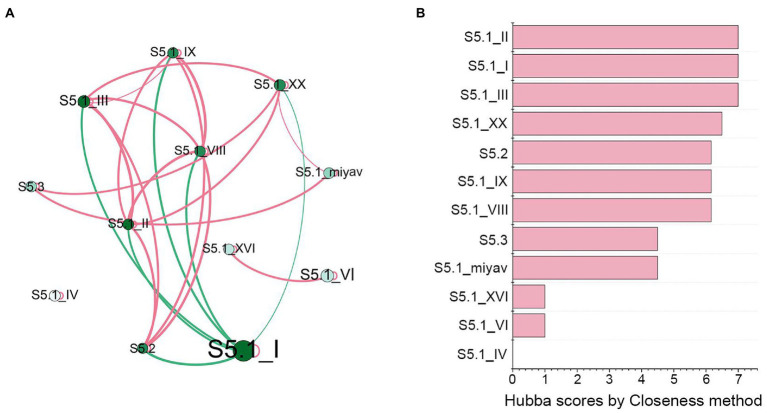
Co-occurrence patterns of *Synechococcus* genotypes. **(A)** The co-occurrence network of *Synechococcus* clades. The size of node represents its average percentage in all samples; the lightness of node represents its degree; the thickness of the edge represents its weight; the red edges represent positive correlations, and the green edges represent negative correlations. **(B)** Hubba scores ranked by the Closeness method represent the significance of each node.

In the co-occurrence network (Figure 4), *Synechococcus* S5.1 clades I, II, and III were the most widely correlated, associating with six lineages. The relationships of clade I with other lineages were all negative, while relationships of clades II or III with other lineages were mostly positive. Hubba scores ranked using the Closeness method revealed that the score of these three lineages was the highest, reaching the value of 7, which further demonstrated their importance in *Synechococcus* assemblage. In contrast, although *Synechococcus* S5.1 clades XVI and VI correlated significantly with each other, they did not associate with any other lineage. Furthermore, *Synechococcus* S5.1 clade IV was separated from the network and was not associated with any lineage.

### Environmental Constraints of *Synechococcus* Assemblages

**Figure 5:**
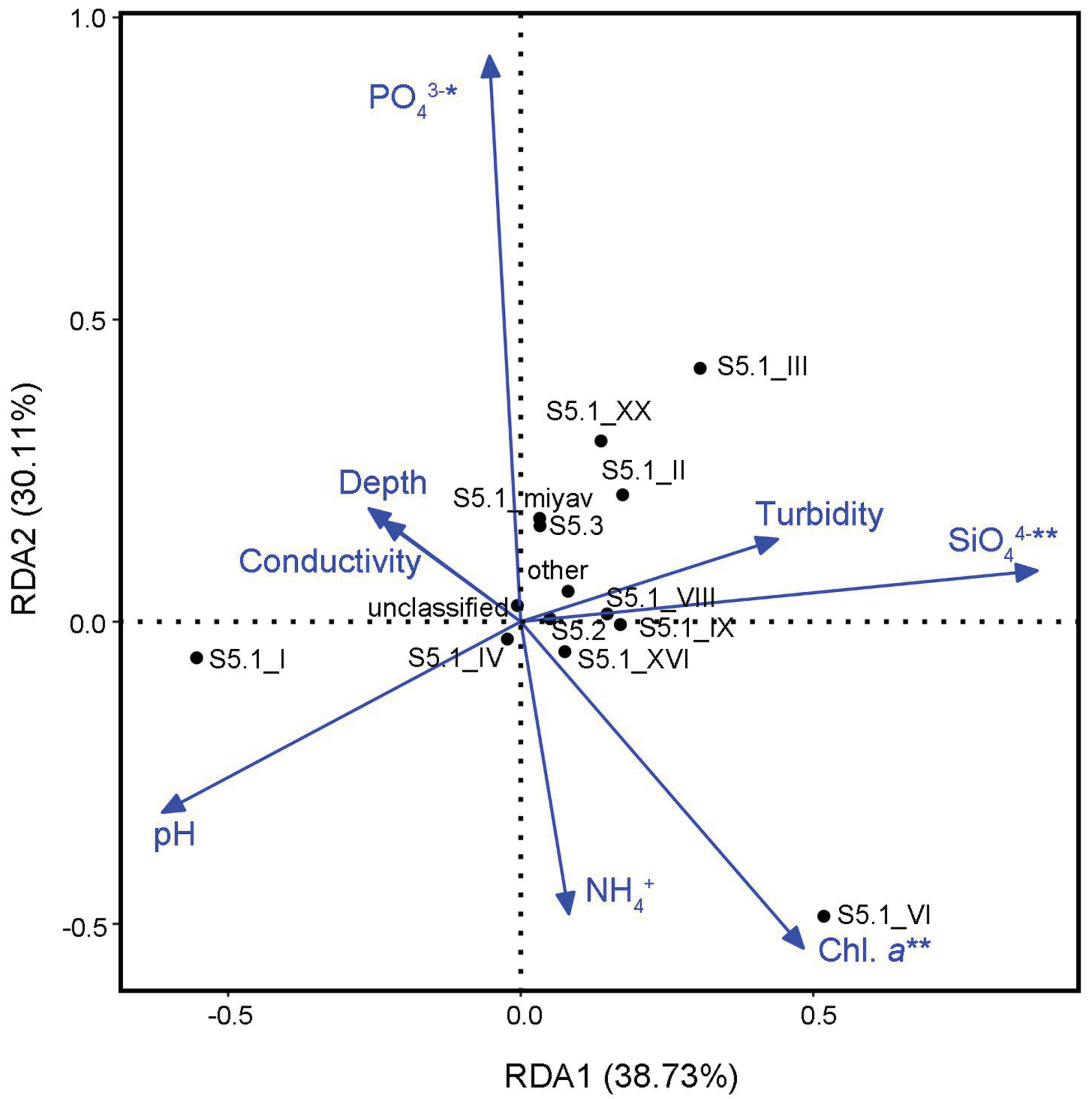
Redundancy analysis (RDA) with the permutation test between *Synechococcus* clades and environmental variables.

The analysis of VIF filtered out five environmental variables, including concentrations of NO_2_^−^ and NO_3_^−^, salinity, temperature, and oxygen saturation. The remaining eight factors totally constrained 77.08% community variance of *Synechococcus* assemblage (*p* < 0.05; Figure 5). According to the permutation test, concentrations of SiO_4_^4−^, PO_4_^3−^, and chl. *a* were significant factors that correlated with the *Synechococcus* composition. Among them, SiO_4_^4−^ concentration was the key constraint variable (*R*^2^ = 0.80, *p* < 0.01).

### Random Forest Analysis Between Each *Synechococcus* Clade and Environmental Variables

**Figure 6:**
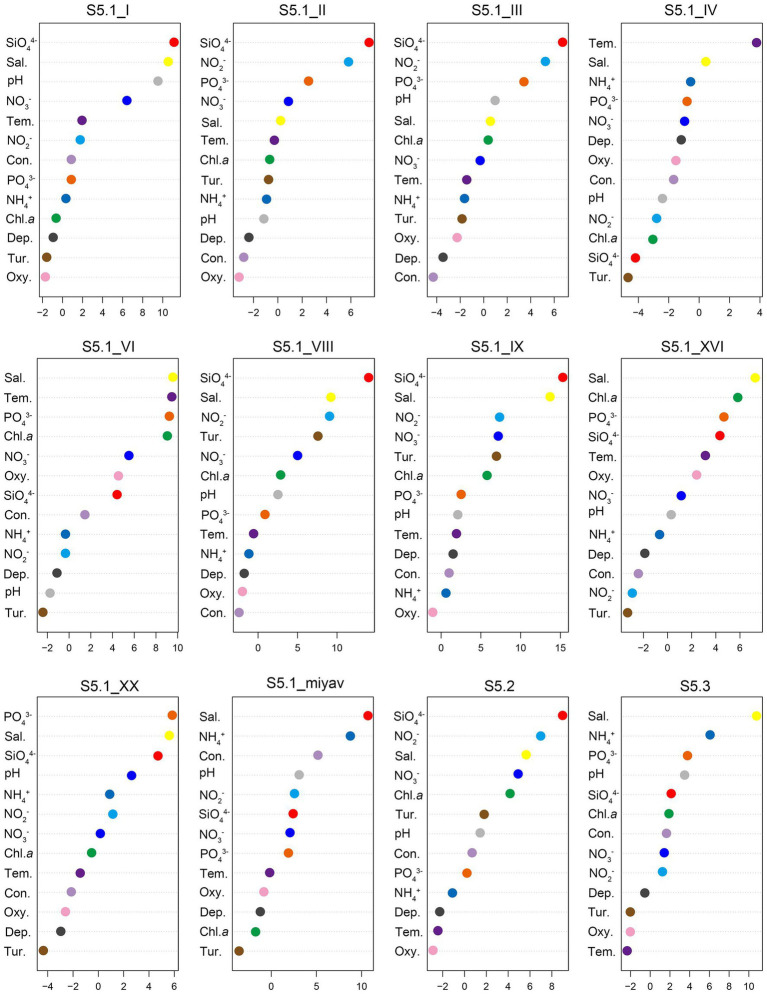
Random forest (RF) analysis between each *Synechococcus* clade and environmental variables. Sal, salinity; Tem, temperature; Con, conductivity; Dep, depth; Tur, turbidity; and Oxy, oxygen saturation. Abscissas are %ImcMSE evaluating the materiality of environmental variables to each *Synechococcus* clade in RF.

Random forest analysis revealed that SiO_4_^4−^ concentration was the most significant variable affecting the distribution of six *Synechococcus* clades, covering S5.1 clade I, clade II, clade III, clade VIII, and clade IX, as well as S5.2 (Figure 6). Salinity was the most significant variable that affected the distribution of four *Synechococcus* clades, including S5.1 clade VI, clade XVI, and clade miyav, as well as S5.3. The temperature was the most significant variable that affected the distribution of *Synechococcus* S5.1 clade IV.

### Linear Regression Analysis Between *Synechococcus* Genotypes and Environmental Variables

Linear OLS regression showed that except for clade IV, significant linear relationships were found between each *Synechococcus* clade and at least one environmental parameter (Table 3). Among them, clade VI related to the most environmental parameters with up to six. Clades I, IX, and S5.2 were next and correlated with five environmental parameters. Clades VIII, XVI, and II correlated with four, three, and two parameters, respectively, and the rest of the four clades correlated only with one parameter. Values of *R*^2^ greater than 0.6 were defined as a strong correlation (Supplementary Figure S4). Clade I correlated strongly to SiO_4_^4−^ concentration (negatively); clade VI correlated strongly with chl. *a* and temperature (both positively); clade VIII correlated strongly with NO_2_^−^ concentration (positively). Clade IX correlated strongly with four parameters, namely, the concentrations of NO_2_^−^ and SiO_4_^4−^, salinity, and turbidity. Among them, the correlation between clade IX and salinity was negative, while that between clade IX and the other three parameters was positive.

**Table 3:** Results of linear ordinary least square (OLS) regression for the relative abundances of *Synechococcus* genotypes and environmental variables.

	I	II	III	IV	VI	VIII	IX	XVI	XX	miyav	S5.2	S5.3
NO_2_^−^	0.35^*^	0.57^**^	0.47^**^			0.68^**^	0.66^**^				0.49^**^	
NO_3_^−^	0.39^*^				0.48^**^		0.35^*^	0.33^*^			0.30^*^	
NH_4_^+^										0.34^*^		0.32^*^
PO_4_^3−^					0.41^*^				0.40^*^			
SiO_4_^4−^	0.70^**^					0.50^**^	0.69^**^				0.44^**^	
Chl.*a*					0.70^**^							
Conductivity												
Salinity	0.51^**^				0.55^**^	0.40^*^	0.63^**^	0.47^**^			0.43^*^	
Temperature					0.64^**^			0.36^*^				
Turbidity		0.41^*^				0.50^**^	0.64^**^				0.29^*^	
pH	0.43^*^											
Oxygen saturation					0.34^*^							

OLS, linear ordinary least squares.

“^*^” represents *p* < 0.05.

“^**^” represents *p* < 0.01.

## Discussion

### Shift in Hydrologic Conditions in Bohai Sea

The development of agriculture, aquaculture, and industry has increased nutrient inputs from land to sea water, which has changed the hydrological conditions in the Bohai Sea (Wang et al., 2018b). It is believed that a gradual change from nitrogen limitation to phosphate and silicate limitations has occurred here (Yu, 2000). We observed that the concentration of NO_3_^−^ in 2019 is higher than that in 2014 (Wang et al., 2018b), which is consistent with the existing consensus that the dissolved inorganic nitrogen (DIN) concentration in the Bohai Sea increased continuously (Su et al., 2011; Wang et al., 2018b). PO_4_^4−^ and SiO_4_^4−^ concentrations were thought to be on a decline in the Bohai Sea (Ning et al., 2010). In comparison, we observed an increase in PO_4_^4−^ concentration but a decrease in SiO_4_^4−^ concentration compared with previous surveys (Wang et al., 2018b). In addition, we found that the ratio of SiO_4_^4−^/DIN was lower than 1 in all samples except those from BS-W. Silicate limitation appears to have become a main hydrological characteristic in the Bohai Sea. According to our record in the Bohai Sea, the environmental conditions exhibited stronger variation between strait and bay areas than variation between June and November as well as different vertical water layers. Poor water exchange and continuous inflow of pollutants resulted in higher concentrations of various nutrients in the LZB than in BS (Su et al., 2011). Considering that environmental changes will stimulate the preferential growth of certain phytoplanktons under suitable conditions and lead to further changes in their communities, we expected the formation of a special phylogenetic composition of *Synechococcus* assemblage in the Bohai Sea, which was confirmed in this study (Ferreira et al., 2007; Yunev et al., 2007; Yan et al., 2020).

### Diversity of *Synechococcus* Assemblages in the Bohai Sea

High-throughput sequencing of *rpo*C1 led to the detection of 12 clades representing *Synechococcus* S5.1, S5.2, and S5.3 (Figure 3). In comparison, previous studies found about 6 to 13 lineages in most of the sea areas of the world, such as open sea and coast of Pacific Ocean (Tai and Palenik, 2009), Gulf of Aqaba (Post et al., 2011), Chesapeake Bay (Chen et al., 2006), Sargasso Sea (Ahlgren and Rocap, 2006), and East China Sea (Choi et al., 2013b). More lineages were identified in estuarine waters, which included freshwater *Synechococcus* and *Cyanobium* species. For example, in total, 17 *Synechococcus* clades were reported in the estuarine and coastal waters of Hong Kong (Xia et al., 2015). Our results suggested that the phylogenetic diversity of *Synechococcus* lineages in the bay and strait areas of the Bohai Sea was similar to that in most worldwide seas, but is lower than that in estuarine waters.

### Phylogeography of *Synechococcus* Assemblages in the Bohai Sea

*Synechococcus* clade I was the absolute dominant lineage in bay and strait areas of the Bohai Sea (Figure 3). *Synechococcus* clades VI and III were also highly abundant genotypes, while their average proportion in all samples was only about one-sixth of that of clade I. *Synechococcus* composition at station BS was similar to that in the adjacent north Yellow Sea (clade I was also found to be dominant), which is probably due to the water quality (Li et al., 2021). However, in sea areas that are farther away, such as south Yellow Sea, East China Sea, and South China Sea, the composition of *Synechococcus* varied highly (Jing and Liu, 2012; Chung et al., 2015; Xia et al., 2017b). In south Yellow Sea, *Synechococcus* clades VI, II, and III were relatively abundant (Xia et al., 2017b). In addition, *Synechococcus* clade II was dominant in the East China Sea and the South China Sea (Xia et al., 2017b).

Compared with other continental seas in the world, the phylogenetic composition of the *Synechococcus* assemblage in the bay and strait areas of the Bohai Sea resembled that in the Sea of Okhotsk (Jing et al., 2009a) and the northwestern waters of the Gulf of Lions (Mella-Flores et al., 2011). In the Sea of Okhotsk, the *Synechococcus* community was composed exclusively of S5.1 and dominated by clades I and IV (Jing et al., 2009a). In the Mediterranean Sea, the *Synechococcus* assemblage was dominated by clades I, III, and IV in the northwestern waters of the Gulf of Lions (Mella-Flores et al., 2011). In contrast, *Synechococcus* assemblage was dominated by a single clade, clade II, with the exception of a peak of clade III in June in the Gulf of Aqaba, Red Sea (Fuller et al., 2005). In the Mediterranean Sea, except for the northwestern waters of the Gulf of Lions, *Synechococcus* assemblage was dominated by groups genetically related to clades WPC1 and VI (Mella-Flores et al., 2011).

Geographical variation in *Synechococcus* composition between BS and LZB in the Bohai Sea was demonstrated (Figures 2, 3). In BS, the composition of *Synechococcus* genotype was similar to that found in open and high-latitude oceans, such as the North Atlantic, southern Bering, and northwestern Pacific Ocean (Huang et al., 2012; Sohm et al., 2016; Xia et al., 2017b). On the contrary, *Synechococcus* assemblage in LZB with higher phylogenetic diversity was mostly composed of clades VI and III, similar to that in coastal waters of Hong Kong and the Pearl River estuary (Xia et al., 2015, 2017a). This may be because of the relatively high nutritional condition of LZB caused by the discharge of exogenous substances from the mainland (Lü et al., 2015). However, the community structure of the *Synechococcus* assemblage did not vary among the vertical water layers in the Bohai Sea. This could be because the Bohai as a marginal sea of continental shelf possesses shallow water depth (the average depth is 18 m) with intense vertical water mixing. Overall, our study supports the conclusion that in addition to geographical proximity, *Synechococcus* phylogeography is driven by environmental conditions (Zwirglmaier et al., 2008).

*Synechococcus* clade VI was the third abundant clade in the bay and strait areas of the Bohai Sea (Figure 3). In particular, its proportion can reach 70% of *Synechococcus* assemblage in LZB-S. However, according to the co-occurrence network, its Hubba score was as low as 1 (Figure 4), representing a low contribution to *Synechococcus* community. This result might suggest that this clade showed low metabolic activity in the Bohai Sea. However, further studies at the transcriptional level are needed to confirm it.

### Variations of *Synechococcus* Assemblages in the Bohai Sea Between June and November

The geographical features, as well as periodical discharges of pollution and freshwater from the continent, could together lead to the strong seasonal variation of *Synechococcus* in the Bohai Sea (Wang et al., 2021). *Synechococcus* S5.1 clades II, III, XX, and miyav appeared only in November. In the global oceans, clade II was the most ubiquitous clade dominant in the oligotrophic open ocean (Sohm et al., 2016). However, consistent with our results, with the increase in latitude, it was gradually replaced by clades I and IV above 37°N. Clade III appeared in the Mediterranean Sea and the Gulf of Mexico before (Farrant et al., 2016). As suggested by a year-round survey in the Red Sea, this clade is also found subject to seasonality, which is abundant during specific months and remains abundantly low over the rest of the year (Post et al., 2011). Clade miyav, represented by strain *Synechococcus* sp. strain miyav, was detected and defined in estuarine waters and coastal waters of Hong Kong (Xia et al., 2015). Similar to our results, this clade mainly occurred in winter. It should be noted that the seasonal variation of *Synechococcus* assemblage was only studied in June (representing summer) and in November (representing winter) due to the cruise limitation in this study. The characteristics of *Synechococcus* assemblage in spring and autumn need further study to clear.

### Correlation Between Environmental Variables and *Synechococcus* Genotypes in the Bohai Sea

Environmental variables affect the community characteristics and structure of *Synechococcus* assemblage in specific environments (Xia et al., 2015). In wide global sea areas, the distribution of *Synechococcus* clades is decided by the oceanic condition defined by temperature, macronutrients, and iron (Sohm et al., 2016). The first axis was mainly a phosphate gradient, whereas the second axis was mainly nitrate concentration and biomass (chl. *a*) gradients in the RDA of the Gulf of Aqaba (Fuller et al., 2005). In contrast, *Synechococcus* assemblage in coastal and estuarine waters appeared to be influenced by physical marine environmental factors. In estuarine and coastal waters of Hong Kong, salinity and temperature significantly affected *Synechococcus* assemblage composition and accounted for 32.0 and 22.0% variance of the *Synechococcus* assemblages, respectively (Xia et al., 2015). However, concentrations of SiO_4_^4−^, PO_4_^3−^, and chl. *a*, were significant variables that correlated with the *Synechococcus* composition in the bay and strait areas of the Bohai Sea (Figure 5). This indicated that in addition to environmental pollution caused by human activities, other biological activities affect the *Synechococcus* assemblage in the environment.

In particular, each of the *Synechococcus* genotypes was linked to different environmental parameters and occupied special ecological niches. Clade I dominated the cold, high-latitude, and nutrient-rich waters; clade III was dominant in warm, oligotrophic, open-ocean habitats in the tropical and subtropical oceans; clade VI became apparent during the transition periods between mixing and stratification (Post et al., 2011; Sohm et al., 2016). According to the RF models, these dominant clades in the bay and strait areas of the Bohai Sea were considerably affected by SiO_4_^4−^ concentration and salinity (Figure 6). Baines et al. (2012) discovered the accumulation of silicon (Si) by *Synechococcus*; however, a clear role of Si in *Synechococcus* has not been revealed. Researchers have shown that Si uptake may be associated with the acquisition of phosphate and that Si accumulation by *Synechococcus* is inadvertent (Brzezinski et al., 2017). Our finding that the concentration of SiO_4_^4−^ was the most significant variable affecting the distribution of six *Synechococcus* clades contradicts the above conclusion of inadvertent accumulation of Si. It was also confirmed by the observation of Wei et al. (2019), which demonstrated that *Synechococcus* biomass including cellular sizes was favored by the dissolved Si (DSi) concentrations. Recent studies have demonstrated that picoplankton size populations, especially *Synechococcus*, contribute a measurable and significant proportion of total biogenic silica (bSi; Wei et al., 2021a). Meanwhile, it was found that atomically dense structures from *Synechococcus* cell lysis were enriched in Si element (Wei et al., 2021b). These signs all indicate the close relationship between *Synechococcus* and Si. In summary, our result demonstrated that Si may play a more important role in each *Synechococcus* genotype, which would add a new dimension to their nutrient physiology and to the suite of resources influencing *Synechococcus* abundance and community structure in nature, although further physiological and genomic studies are required to verify it.

*Synechococcus* can be considered a key environmental indicator. Rajaneesh et al. (2015) initially constructed the indicator relationship between two kinds of *Synechococcus* (phycoerythrin-rich *Synechococcus* and phycocyanin-rich *Synechococcus*), as well as between the trophic status index and various environmental parameters in the Cochin backwaters, west coast of India, and found the superiority of *Synechococcus* as an indicator organism. However, considering its high phylogenetic diversity and correlations with various environmental variables, their conclusion still underestimated the enormous potential of *Synechococcus* in indicating the marine environment. Here, we identified a rich linear correlation between *Synechococcus* clades and environmental parameters in the bay and strait areas of the Bohai Sea (Table 3; Supplementary Figure S4). A significant linear relationship existed between each of the 11 *Synechococcus* clades and at least one environmental parameter. In particular, clade VI correlated with the most parameters, reaching six. By expanding the number of samples and increasing the density of sampling sites, more stable equations regarding the relation between the *Synechococcus* clades and the environmental parameters can be achieved, which is our next research focus.

## Conclusion

Continental seas, affected strongly by human activities, possess dynamic environmental conditions. In the Bohai Sea, the largest continental sea of China, silicate limitations appear to be a main hydrological characteristic. Because of this, a special phylogenetic composition pattern of *Synechococcus* assemblage was formed in this sea area. High-throughput sequencing of *rpo*C1 revealed that *Synechococcus* diversity in the bay and strait areas of the Bohai Sea was similar to that in most seas worldwide, but was lower than that in estuarine waters. Geographical variations in *Synechococcus* composition were observed between BS and LZB. Obligately dominated by clade I, the composition of *Synechococcus* genotype in BS was similar to that in open and high-latitude oceans. On the contrary, the *Synechococcus* assemblage in LZB, with higher phylogenetic diversity and highly abundant clades VI and III, was similar to that in coastal and estuarine waters. *Synechococcus* S5.1 clades II, III, XX, and miyav only appeared in winter. The community structure of the *Synechococcus* assemblage did not vary among the vertical water layers, which could be because of its shallow water depth with intense vertical water mixing. Silicate is a key environmental variable that affects the community characteristics and structure of the *Synechococcus* assemblage in the bay and strait areas of the Bohai Sea, probably because of silicon limitations. This may change the previous conclusion that silicate accumulation by *Synechococcus* is inadvertent, although further physiological and genomic studies are required for verifying this.

## Data Availability Statement

The datasets presented in this study can be found in online repositories. The names of the repository/repositories and accession number(s) can be found at: https://www.ncbi.nlm.nih.gov/, SRR15404031—SRR15404044.

## Author Contributions

SQ and JL: conceptualization and writing—review and editing. JL and TW: methodology and investigation. TW and XC: data curation and visualization. TW: writing—original draft preparation. SQ: supervision. JL: project administration and funding acquisition. All authors contributed to the article and approved the submitted version.

## Funding

This study was financially supported by the Key Deployment Project of Centre for Ocean Mega-Science, Chinese Academy of Sciences (COMS2020Q09), the National Key Research and Development Program of China (No. 2018YFD0901102), and the National Natural Science Foundation of China (No. 42176131).

## Conflict of Interest

The authors declare that the research was conducted in the absence of any commercial or financial relationships that could be construed as a potential conflict of interest.

## Publisher’s Note

All claims expressed in this article are solely those of the authors and do not necessarily represent those of their affiliated organizations, or those of the publisher, the editors and the reviewers. Any product that may be evaluated in this article, or claim that may be made by its manufacturer, is not guaranteed or endorsed by the publisher.
