# Supplementary material for: Phylogenetic Structure of Synechococcus Assemblages and Its Environmental Determinants in the Bay and Strait Areas of a Continental Sea
Source: Front Microbiol. 2022 Apr 6;13:757896. doi: 10.3389/fmicb.2022.757896 (PMC9020259; doi:10.3389/fmicb.2022.757896)
Supplement: Supplementary file 1 [file Data_Sheet_1.docx]

Supplementary Material

# Supplementary Figures and Tables

## Supplementary Figures


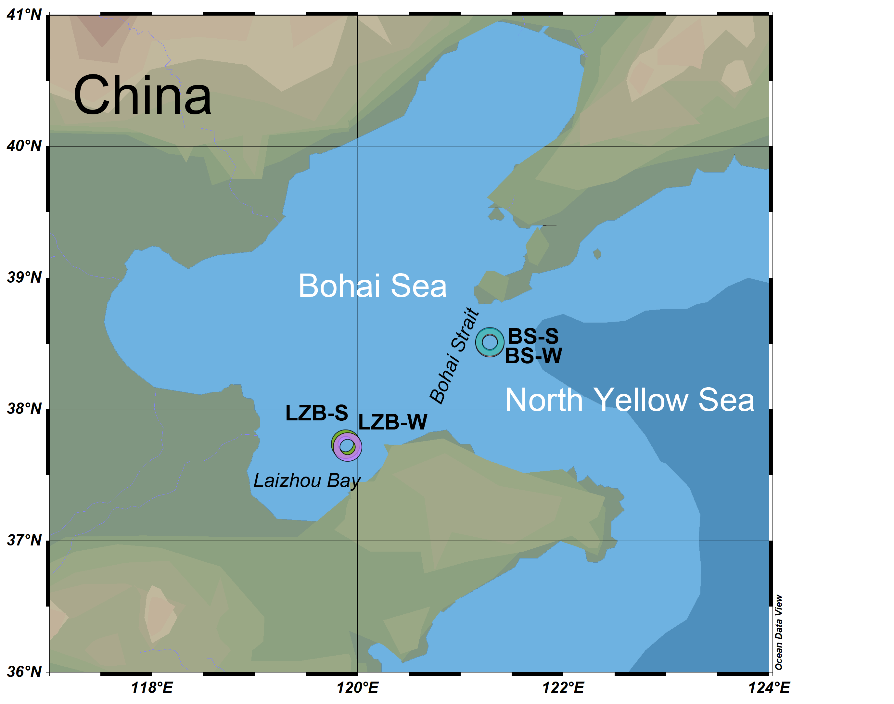


**Supplementary Figure 1.** Geographic positions of sampling stations in two cruises. BS-S: samples collected at station BS in summer (June); BS-W: samples collected at station BS in winter (November); LZB-S: samples collected at station LZB in summer (June); LZB-W: samples collected at station LZB in winter (November).


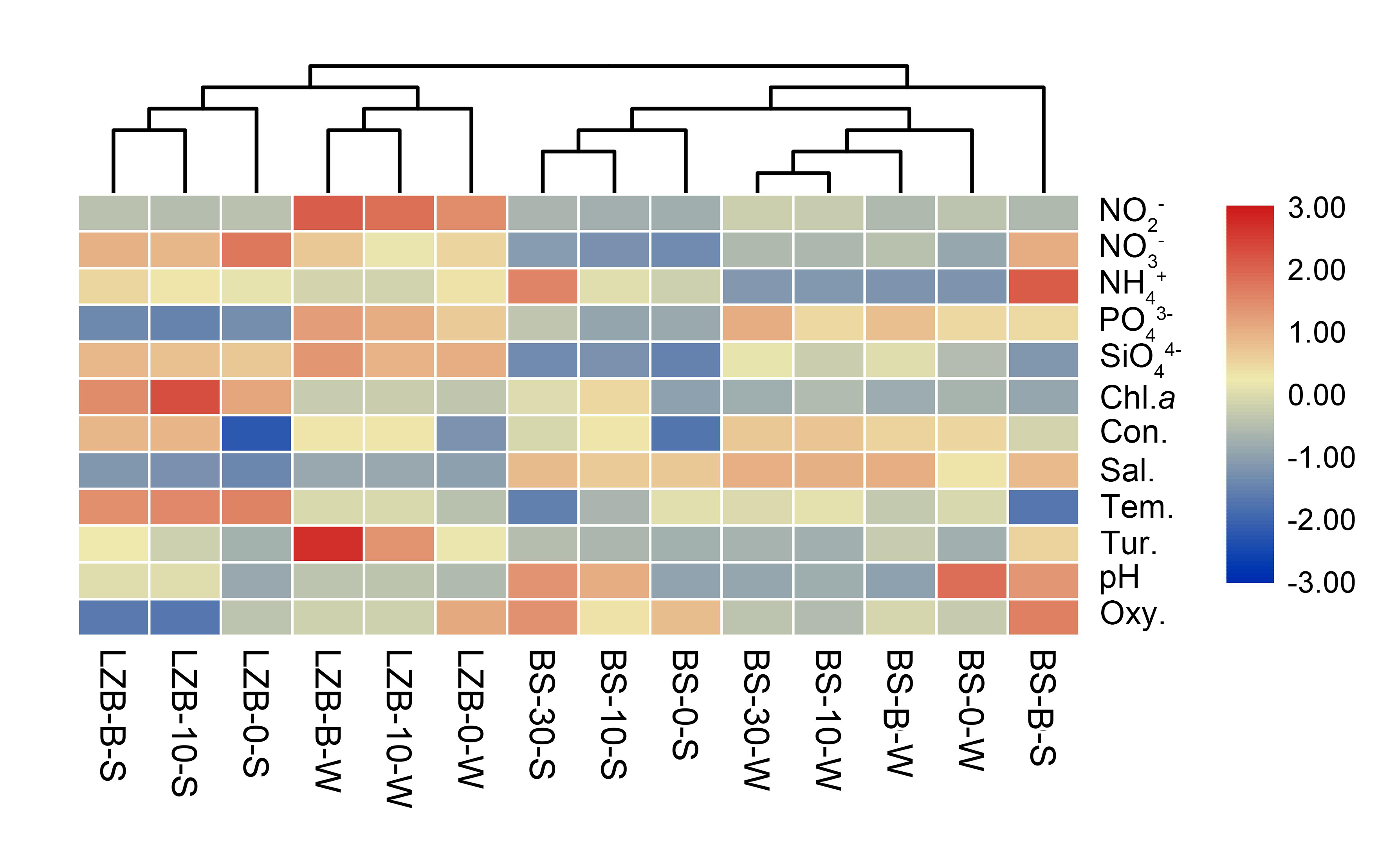


**Supplementary Figure 2.** Heatmap of scaled environmental variables with clustering of samples. Sal: salinity; Tem: temperature; Con: conductivity; Dep: depth; Tur: turbidity; Oxy: oxygen saturation.


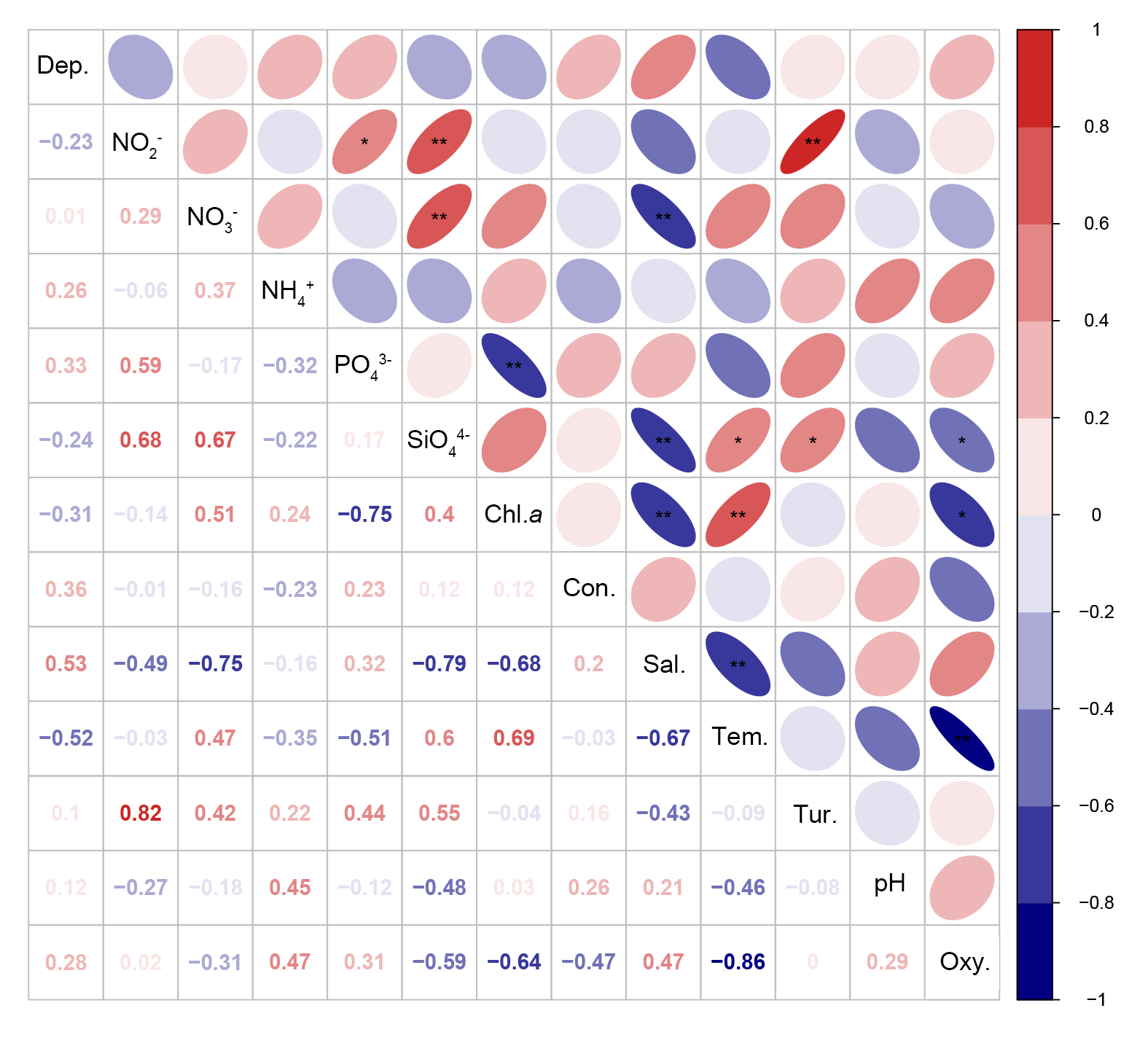


**Supplementary Figure 3.** Spearman correlations among environmental variables. Sal: salinity; Tem: temperature; Con: conductivity; Dep: depth; Tur: turbidity; Oxy: oxygen saturation.


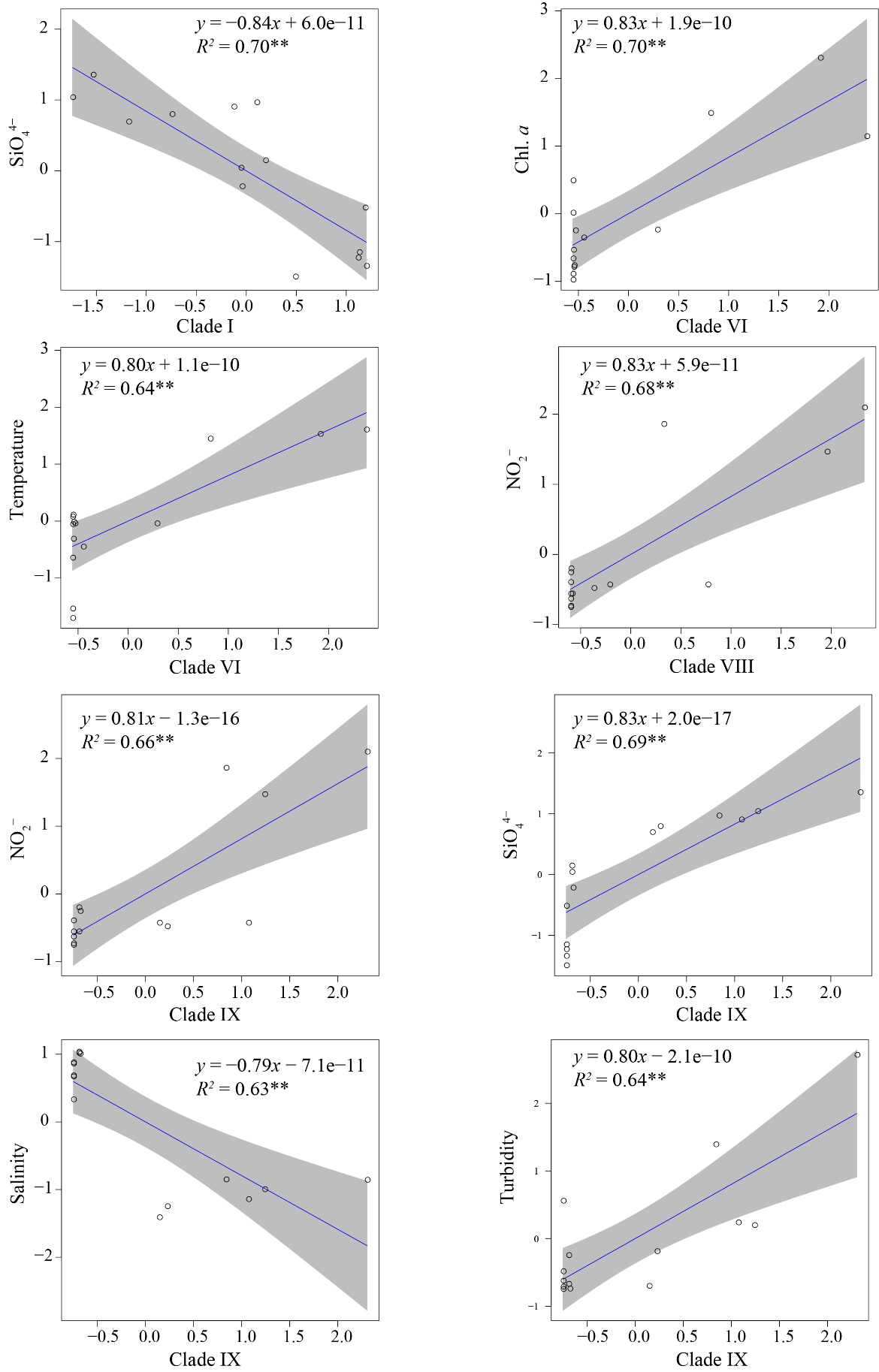


**Supplementary Figure 4.** Linear OLS regression analysis of *Synechococcus* genotypes and environmental variables. Only OLS regression with *R^2^* > 0.6 was exhibited in the figure. OLS: linear ordinary least squares.
